# Detection of weakly conserved ancestral mammalian regulatory sequences by primate comparisons

**DOI:** 10.1186/gb-2007-8-1-r1

**Published:** 2007-01-03

**Authors:** Qian-fei Wang, Shyam Prabhakar, Sumita Chanan, Jan-Fang Cheng, Edward M Rubin, Dario Boffelli

**Affiliations:** 1 Genomics Division, Lawrence Berkeley National Laboratory, Berkeley, California 94720 USA; 2 US Department of Energy Joint Genome Institute, Walnut Creek, California 94598, USA; 3 Current address: Section of Hematology/Oncology, Department of Medicine, University of Chicago, 5814 S. Ellis Avenue, Chicago, IL 60637, USA

## Abstract

**Background:**

Genomic comparisons between human and distant, non-primate mammals are commonly used to identify *cis*-regulatory elements based on constrained sequence evolution. However, these methods fail to detect functional elements that are too weakly conserved among mammals to distinguish them from non-functional DNA.

**Results:**

To evaluate a strategy for large scale genome annotation that is complementary to the commonly used distal species comparisons, we explored the potential of deep intra-primate sequence comparisons. We sequenced the orthologs of 558 kb of human genomic sequence, covering multiple loci involved in cholesterol homeostasis, in 6 non-human primates. Our analysis identified six non-coding DNA elements displaying significant conservation among primates but undetectable in more distant comparisons. *In vitro *and *in vivo *tests revealed that at least three of these six elements have regulatory function. Notably, the mouse orthologs of these three functional human sequences had regulatory activity despite their lack of significant sequence conservation, indicating that they are ancestral mammalian *cis*-regulatory elements. These regulatory elements could be detected even in a smaller set of three primate species including human, rhesus and marmoset.

**Conclusion:**

We have demonstrated that intra-primate sequence comparisons can be used to identify functional modules in large genomic regions, including *cis*-regulatory elements that are not detectable through comparison with non-mammalian genomes. With the available human and rhesus genomes and that of marmoset, which is being actively sequenced, this strategy can be extended to the whole genome in the near future.

## Abstract 2

Intra-primate genome comparisons identify functional modules that are undetected by comparisons with non-mammalian genomes.

## Background

Identifying *cis*-regulatory elements in the human genome, such as promoters and enhancers that regulate gene expression in normal and diseased cells and tissues, is a major challenge of the post-genomic era. Inter-species sequence comparisons have emerged as a major technique for identifying human regulatory elements, particularly those to the sequenced mouse, chicken and fish genomes [1]. However, a significant fraction of empirically defined human regulatory modules are too weakly conserved in other mammalian genomes, such as the mouse, to distinguish them from non-functional DNA [2], and are completely undetectable in non-mammalian genomes [3,4]. Identification of such significantly divergent functional sequences will require complementary methods in order to complete the functional annotation of the human genome.

Deep intra-primate sequence comparison, referred to as 'phylogenetic shadowing', is a novel alternative to the commonly used distant species comparisons [5]. However, primate shadowing has so far only been applied to the identification of novel *cis-*regulatory elements in short, targeted genomic fragments (≤ 2.0 kb) [6,7], due to the lack of sequence data from multiple primates. Thus, it remains to be determined if this approach is useful in identifying otherwise undetectable regulatory regions in unbiased scans of large genomic loci. Perhaps for this reason, primate shadowing has been almost entirely overlooked as a predictor of regulatory elements.

Here we evaluate the possibility of using deep primate sequence comparisons in large genomic regions (approximately 100 kb) to systematically uncover *cis*-regulatory elements that are undetectable through mammalian or more distant comparisons. We focused on genes involved in cholesterol metabolism, since this is a physiological process marked by numerous differences between human and distant mammals. In particular, differential regulation of LXRα and its target genes is thought to contribute to inter-species variation in the plasma cholesterol response to dietary cholesterol intake [8]. We evaluated the sensitivity and true positive rate of primate shadowing using as a test set known functional sequences in eight loci, for which we sequenced a phylogenetically representative panel of primate species. Using a combination of close and distant species comparisons, we then identified six human sequences characterized by primate-specific conservation in these eight gene loci, and tested them for enhancer function *in vitro *and *in vivo*. Finally, we determined if a subset of primate sequences comprising genomes currently available or being sequenced would suffice to identify divergent mammalian regulatory sequences.

## Results

### Primate comparison identifies known functional sequences in large genomic intervals

To test the power of primate shadowing to identify functional elements in large genomic intervals, we sequenced the primate orthologs of eight human loci containing *LXRα *and eight of its target genes: *SREBF1*, *CYP7A1*, *LDLR*, *ABCG5*, *ABCG8*, *APOE *cluster, *APOCIII *cluster, and *HMGCR*. The sequenced species comprised six anthropoid primates (baboon, colobus, dusky titi, marmoset, owl monkey and squirrel monkey) and one prosimian (lemur). The targeted genomic segments included all exons, introns and flanking intergenic regions of the above mentioned genes, encompassing 558 Kb of human genomic DNA.

**Figure 1:**
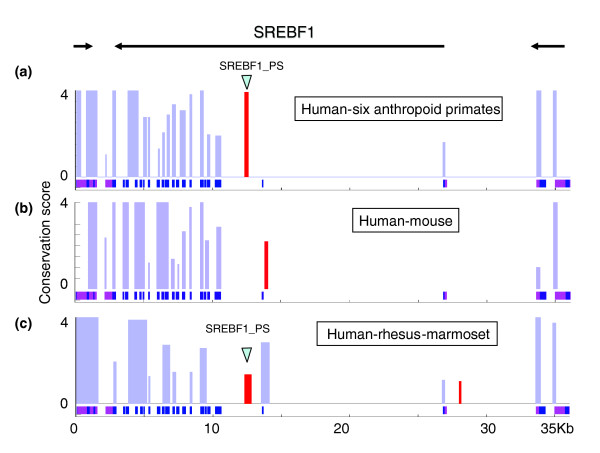
Conservation profiles of a representative region, the *SREBF1 *locus, using close (primate) and distant (human-mouse) species comparisons. **(a) **Seven-primate (human, baboon, colobus, marmoset, dusky titi, owl monkey, and squirrel monkey), **(b) **human-mouse, and **(c) **three-primate (human, rhesus, and marmoset) conservation profiles in the *SREBF1 *locus with flanking genes partially shown. Sequence conservation was calculated using Gumby and visualized using RankVISTA with the human sequence as reference. Vertical bars above the horizontal axis depict evolutionarily conserved sequences, with height indicating the conservation score (-log(conservation *p *value); see Materials and methods). Coding exons (dark blue) and untranslated regions (UTRs; magenta) are marked below the horizontal axis. Vertical bars that overlap coding exons or UTRs are colored light blue, while non-overlapping bars are colored red. The arrowhead denotes SREBF1_PS, a non-coding element conserved in primates (*p *value ≤ 0.005) but not in the mouse (*p *value > 0.1).

**Figure 2:**
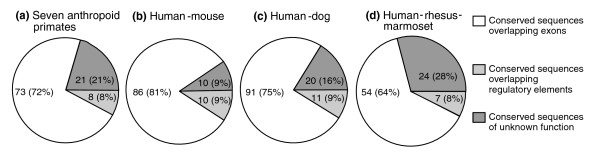
Primate comparisons identify known functional elements and conserved non-coding sequences in genomic intervals encompassing *LXRα*, *SREBF1*, *CYP7A1*, *LDLR*, *ABCG5*, *ABCG8*, *APOE *cluster, *APOCIII *cluster, and *HMGCR*. The number of evolutionarily conserved sequences (*p *value ≤ 0.1) overlapping exons, previously known regulatory elements and unannotated regions (new predictions) in the eight loci are shown for the following species sets: **(a) **seven anthropoid primates, **(b) **human-mouse, **(c) **human-dog and **(d) **human-rhesus-marmoset. Percentages were calculated by dividing the number of conserved sequences of each type by the total number of conserved elements (× 100).

We identified sequences evolutionarily conserved among seven anthropoid primates (the six targeted anthropoids plus human) or among all eight primates (anthropoid plus lemur) using Gumby, an algorithm that detects sequence blocks evolving significantly more slowly than the local neutral rate [3,9,10]. Most of the conserved regions overlapped exons (see, for example, Figure 1). The true-positive rate, defined as the fraction of conserved regions overlapping exons or known regulatory regions, was 80% in the 7-primate comparison (Figure 2a) and 81% using the 8-primate set (data not shown). The human-dog comparison, which approximately matches the combined branch length of the primate comparison, had a similar true-positive rate of 84% (Figure 2c). The more distant human-mouse comparison displayed a marginally higher true-positive rate of 90% (Figure 2b). This is consistent with the theoretical prediction that statistical power increases with the total branch length of the species set [11]. It should be noted, though, that regulatory sequence annotation of the eight loci we analyzed is probably highly incomplete. Therefore, these true-positive rates are lower bounds; some or all of the 'false positives' could eventually be reassigned as true positives upon expansion of the set of sequences annotated as *cis*-regulatory. Thus, due to incomplete annotation of functional elements, it is not clear if the difference between the primate and human-mouse true positive rates reflects a significant difference in reliability between the two sets of predictions. On average, 64% of the exons in the 8 loci overlapped conserved regions in both the 7-primate and 8-primate comparisons. Similar sensitivity was obtained in human-mouse (65%) and human-dog (71%) comparisons. Thus, primate sequence comparison was approximately equivalent to pairwise human-mouse or human dog analysis in identifying exons.

### Phylogenetic shadowing using seven anthropoid primates identifies non-coding sequences with primate-specific conservation

To identify *cis*-regulatory sequences not detectable in comparisons between human and distant mammals, we searched the 8 gene loci for non-coding sequences highly conserved among primates (*p *value ≤ 0.005) but not detectable in human-mouse or human-dog comparisons (*p *value > 0.1). Gumby analysis of human and six other anthropoid primates identified six anthropoid-primate-conserved non-coding regions (Additional data file 4). These sequences were either undetectable (*p *value > 0.1) or less significantly conserved (*p *value > 0.005) when the prosimian lemur was included in the primate set (data not shown).

**Figure 3:**
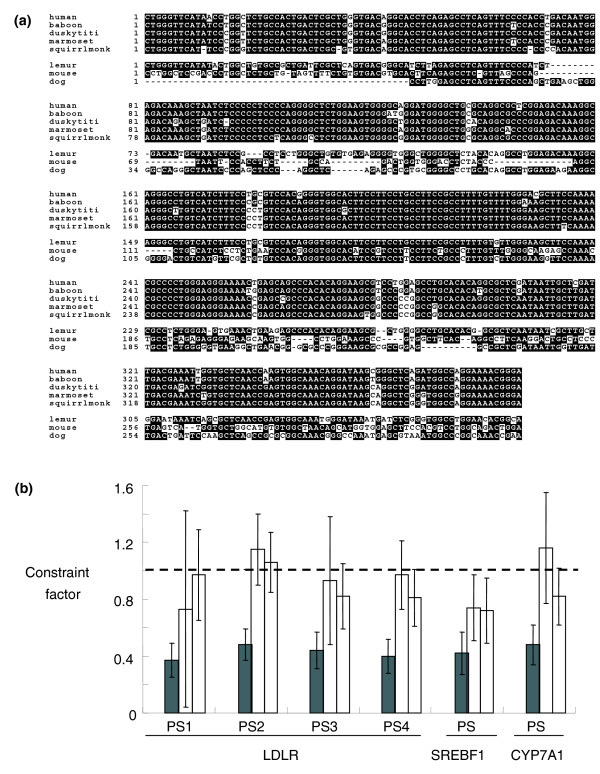
Evolutionary conservation of six primate-conserved sequences in anthropoid primates, but not between human and mouse or dog. **(a) **Sequence alignment of a representative primate-conserved sequence LDLR_PS4. Similar alignments for LDLR_PS2 and SREBF1_PS are provided as Additional data files 1 and 2, respectively. **(b) **The constraint factor of a sequence element is defined as the nucleotide substitution rate (total branch length of the phylogenetic tree) within the element relative to the background non-coding rate in the aligned sequences. Constraint factors in the anthropoid primate comparisons (green bars) are consistently well below one (dashed line). Human-mouse (dotted) and human-dog (white) constraint factor ranges of the six sequences are broader, mostly exceeding one at the upper limit. Error bars indicate 95% confidence intervals.

To independently confirm the (anthropoid) primate-specific nature of sequence conservation in these six regions, we compared their nucleotide substitution rate to that of non-exonic sequences in the same locus. Evolutionarily conserved sequences are defined by a constraint factor (ratio of the substitution rate of test sequences to the non-exonic average) smaller than 1. We found that the six primate-conserved sequences had constraint factors well below 1 among anthropoid primates, as expected, and much closer to 1 in the human-mouse and human-dog comparisons (Figure 3). Finally, none of the 6 sequences overlapped significantly conserved segments identified by the phastCons program [12] in a 17-species alignment of the human genome to (mostly) non-primate mammals and more distant vertebrates [13], which further confirms the primate-specificity of their evolutionary conservation.

### Non-coding sequences with primate-specific conservation include three regulatory elements

**Figure 4:**
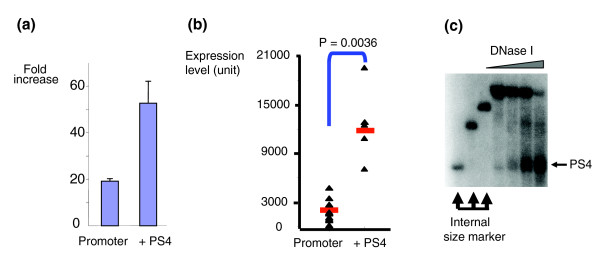
Functional assays indicate enhancer activity for a representative primate-conserved element, human *LDLR *PS4. Luciferase assay analysis of **(a) **transient transfections into human HepG2 cells and **(b) **plasmid DNA transfer into mouse liver. The luciferase reporter constructs tested are either the *LDLR *promoter alone (promoter), or the promoter in combination with the human *LDLR *PS4 (+ PS4). Fold increase over the empty vector is shown. Error bars in (a) indicate standard deviation. Each triangle in (b) represents luciferase activity in an individual mouse. Red bars denote the median activity of each construct. Luciferase activity is reported in arbitrary units. **(c) **DNase I hypersensitive site mapping around the *LDLR *PS4 region in human liver cell line HepG2. Vertical arrows indicates the lane with internal size marker that was generated by enzyme digestion of the *LDLR *PS4 sequence. The hypersensitive site is indicated by a horizontal arrow. Co-migration of the internal size marker with the hypersensitive site localizes the hypersensitive site to the *LDLR *PS4 sequence.

To explore the potential regulatory function of these primate-conserved elements, we examined their ability to drive reporter gene expression in both a transient transfection assay in human HepG2 cells and an *in vivo *mouse liver gene transfer assay. Since it is possible that the computational prediction only captures part of the entire regulatory module, each human element plus 200 to 400 base-pairs (bp) of flanking sequence on either side was cloned upstream of the human promoter of the gene closest to each element and fused to a luciferase reporter gene (see Materials and methods). Therefore, the included flanking sequences may also contribute to the observed regulatory activity. Two elements showed enhancer activity, increasing the expression of a luciferase reporter gene 1.6- to 5-fold in both the human liver cell line HepG2 and *in vivo *in mouse liver, while a third element appeared to be a silencer, suppressing luciferase expression by 50% (Figure 4, Table 1 and data not shown). While a third element, LDLR_PS2, showed modest enhancer activity in HepG2 cells (approximately 1.6-fold increase over promoter alone), its activity in 293T cells was much stronger (approximately 5-fold increase over promoter alone), presumably due to availability in these cells of appropriate transcription factors, such as SREBFs, that are capable of activating *LDLR *[14]. In an independent assay of transcription potential, both enhancer elements were shown to be DNase I hypersensitive sites in HepG2 cells (Figure 4, Table 1 and data not shown), suggesting that the corresponding DNA elements are involved in transcriptional regulation of the endogenous genes. To confirm that the primate orthologs of these identified human regulatory elements are also functional, we cloned the aligned *LDLR *PS4 sequences from baboon, dusky titi, marmoset and lemur into the luciferase reporter vector and tested their ability to drive reporter gene expression in HepG2 cells. All orthologous non-human primate sequences showed enhancer activity (Additional data file 3).

### Regulatory sequences with primate-specific conservation have functional orthologous mammalian counterparts

Since these functional human regulatory elements exhibited primate-specific sequence conservation, we explored whether their functional role is unique to primates. Although human-mouse comparison failed to identify these sequences as constrained (Gumby *p *value > 0.1; Figures 1 and 3b), we were able to identify the aligned counterparts to the three primate-conserved functional sequences in mouse using the global alignment program MLAGAN [15]. To explore the regulatory function, if any, of these mouse orthologs, we cloned the aligned sequences into the luciferase reporter vector described above and compared their activity to that of the human sequence. Despite the lack of statistically significant conservation between the rodent and human sequence, all three mouse orthologs exhibited regulatory activity in the same direction to that observed for the human elements (data not shown). Thus, the silencer and the two enhancers identified through primate-specific sequence conservation are ancestral mammalian regulatory elements, rather than newly evolved functional regions specific to primates.

### A smaller set of three anthropoid primates is sufficient to detect the newly identified regulatory elements

The primate set we used to identify known functional elements and the three divergent mammalian regulatory sequences comprises human, baboon, colobus, marmoset, squirrel monkey and owl monkey. However, it is unlikely that all the corresponding genome sequences will be available in the near future. Of the set of most informative primate genomes for comparative analysis [6], the human and rhesus genome sequences are already publicly available, and the marmoset genome is currently being sequenced. We tested whether comparisons among these three species were sufficient to detect functional sequences in the eight lipid gene loci. Human-rhesus-marmoset comparison identified 55% of the 160 exons (versus 7 primates: 64%) with a true-positive rate of 72% (79% for 7 primates) (Figure 2a,d), suggesting that a significant fraction of exons can be detected using a limited number of primates [16]. We subsequently assessed the ability of human-rhesus-marmoset comparison to detect the three newly identified regulatory sequences. As was observed in the comparison of 7 anthropoid primates, both *LDLR *enhancers were highly conserved (*p *value <0.005) in the 3-way primate analysis and ranked among the three most conserved non-coding sequences in the 75 kb genomic region (data not shown). The smaller set of 3 primates was also sufficient to detect the silencer in the *SREBF1 *locus (non-coding rank 1), though not as strongly (*p *value = 0.044; Figure 1c). The lower statistical significance of the *SREBF1 *silencer in the three-primate analysis relative to the seven-primate analysis is due to the lower combined branch length of the former. These results suggest that availability of the marmoset genome sequence will facilitate genome-wide analysis of primate-specific conservation, and uncover regulatory sequences that are undetectable in distant, non-primate comparisons.

## Discussion

Our analysis of over 500 kb of sequence from each of 7 primate species revealed 6 non-coding elements significantly conserved exclusively in primates, of which 3 were found to have gene regulatory activity in a variety of *in vitro *and *in vivo *assays. These three regulatory sequences are so weakly conserved in distant, non-primate mammals that none of the three independent methods we tested were able to detect them in mammalian comparisons. However, primate-specific conservation does not imply primate-specific function. Since the mouse orthologs are also functional, the identified sequences appear to be ancestral mammalian regulatory elements, as opposed to newly evolved functional sequences specific to primates. Nonetheless, it is likely that primate sequence comparison could also identify the subset of functional sequences that arose after primates split from distant mammals.

Our results do not of course suggest that primate comparison is optimal for detecting all classes of regulatory sequence. If a human regulatory sequence is constrained in all mammals, for example, then multi-mammal species comparison is clearly preferable to primate comparison, since the mammalian species tree has greater combined branch length and, consequently, greater statistical power. However, it is well known that many human regulatory elements show no evidence of constraint in mammalian comparisons [2]. We have demonstrated for the first time that primate comparisons can robustly identify at least some members of this class of 'mammal-diverged' human regulatory sequences, even in large (approximately 100 kb) genomic regions.

It is worth noting that the three detected regulatory elements displayed only marginal sequence conservation in the prosimian lemur. This result suggests that primate comparisons should be limited to anthropoids (old world monkeys and new world monkeys) to sensitively detect divergent mammalian *cis*-regulatory elements. It is not clear at this point how many additional functional non-coding elements could be detected in the human genome on the basis of primate shadowing, relative to the number of elements already identifiable using the available mammalian genome sequences. However, it is encouraging that, at a conservation *p *value threshold of 0.005, primate shadowing expanded the set of predicted non-coding functional elements by 55% (6 elements with primate-specific sequence conservation versus 11 predicted by human-mouse and human-dog) in the 8 loci examined in this study. Further large-scale studies are required to precisely quantify the value added by multiple-primate analysis.

As a consequence of high sequence identity between humans and great apes, our closest relatives, chimpanzee and gorilla, add very little to the power of primate sequence comparisons. The phylogenetically most informative set of primate species includes old world monkeys (for example, rhesus macaque) and new world monkeys (for example, marmoset), in addition to human [6,7]. The three functional sequences revealed by seven-primate comparison were also detectable in the three-way human-rhesus-marmoset analysis, albeit less robustly, due to the shorter combined branch length of the three-way comparisons. Since the human and rhesus genome sequences are already publicly available, and the marmoset genome is currently being sequenced, our results support the feasibility of genome-wide discovery of primate-conserved regulatory elements.

Sequence divergence of the identified regulatory elements between human and distant mammals may reflect functional changes in these sequences. *Cis*-regulatory elements with primate-specific sequence conservation are, therefore, potential substrates for determining the molecular basis of primate-specific aspects of gene expression. Previously, we described gain of sterol responsiveness in the anthropoid primate LDLR_PS2 enhancer [17] (Table 1). It is possible that primate-specific sequence conservation of the other two newly identified regulatory elements also reflects qualitative or quantitative expression differences between primates and non-primate mammals, which might be revealed by further in-depth functional characterization and sequence analysis. On the other hand, it is also possible that the lack of significant sequence conservation of some regulatory elements in distant mammals merely reflects the accumulation of compensatory mutations over tens of millions of years, which would retain functional similarity in the absence of significant sequence similarity [18,19]. Finally, it is possible that short sequence motifs such as transcription factor binding sites within the newly discovered regulatory elements are constrained in all mammals, while the entire elements are significantly conserved only in primates. In one example, we were able to find a conserved functional mammalian AP-4 site in the LDLR_PS2 enhancer [17] (data not shown).

## Conclusion

Our results demonstrate that deep intra-primate sequence comparison can be used to identify functional modules such as exons, enhancers and silencers in large genomic regions. Most importantly, analysis of primate-specific conservation allowed detection of three divergent ancestral *cis*-regulatory elements, which were not detectable by more distant mammalian comparisons. With the availability of multiple primate genomes, it should be possible to improve the functional annotation of the human genome by uncovering numerous such *cis*-regulatory sequences, some of which potentially contribute to gene expression differences between primates and distant mammals.

## Materials and methods

### Sources of sequence and annotation data

Primate bacterial artificial chromosome (BAC) clones containing targeted loci were purchased from Children's Hospital Oakland Research Institute in Oakland, California [20]. Draft sequences of baboon, colobus, dusky titi, marmoset, owl monkey, squirrel monkey, and lemur BACs were determined by sequencing ends of 3 kb subclones to 8- to 10-fold coverage using BigDye terminators (Applied Biosystems, Foster City, CA, USA) and assembling reads into contigs with the Phred-Phrap-Consed suite as described previously [21]. All BAC sequences were submitted to GenBank (see Additional data file 6 for accession numbers). Human, mouse, dog and rhesus sequences were downloaded from the UCSC Genome Bioinformatics worldwide web site [13]. Based on the human March 2006 assembly hg18, the coordinates for the analyzed human loci are: *SREBF1*, chr17:17653939-17690020; *CYP7A1*, chr8: 59525742-59605413; *LDLR*, chr19:11054146-11127904; *ABCG5/ABCG8*, chr2:43835998-43966668; *APOE *cluster, chr19:50080832-50149764; *APOCIII *cluster, chr11:116137283-116217351; *HMGCR*, chr5:74646522-74714122; *LXRα*, chr11:47231580-47253367. Exon annotations of these regions were obtained from the UCSC Genome Bioinformatics website [13]. The promoter sequence of a gene is defined as the 1 kb region upstream of the transcription start site. Four enhancers in the *APOE *locus were previously described [22,23]. These four *APOE *enhancers, together with 15 promoters in the 8 genomic loci, comprise the set of known regulatory regions.

### Analysis of sequence conservation

All sequence alignments were carried out using MLAGAN [15]. Aligned sequences were scanned for statistically significant (*p *value ≤ 0.1) evolutionarily conserved regions using Gumby [3,9,10]. We defined primate-specific conserved elements as those human sequences that were highly conserved (Gumby *p *value ≤ 0.005) among anthropoid primates, but not conserved in more distant mammalian comparisons. Mammalian sequence conservation was defined as: *p *value ≤ 0.1 in human-mouse or human-dog comparison; or 70% human-mouse sequence identity over at least 100 bp [24]; or significant conservation in an alignment of 17 vertebrate genomes [12].

Evolutionarily conserved regions identified by Gumby were visualized using RankVISTA [25]. Conservation scores in the RankVISTA plots were calculated as the negative logarithm of the Gumby *p *value.

The constraint factor of a conserved non-coding sequence (CNS) was defined as the nucleotide substitution rate (summed over all branches of the phylogenetic tree) within the element divided by the local background substitution rate at intronic and intergenic positions in the locus (neutral rate). We estimated substitution rates along each lineage by maximum likelihood using fastDNAml [26].

### Plasmid constructs

The human promoter was cloned in the proper orientation upstream of the luciferase cDNA in the pGL3Basic construct (Promega, Madison, WI, USA). The primate-specific elements from human or mouse were PCR cloned into polylinker sites upstream of the promoter of the closest gene (see Additional data file 5 for primer sequences). Each human element includes the primate-conserved sequence plus approximately 200 to 400 bp of flanking sequence. Thus, approximately 1,000 bp of total sequence are tested in each reporter construct.

### Transient-transfection reporter assay

Cells were grown at 37°C and 5% CO_2 _in the minimum essential medium (ATCC, Manassas, VA, USA) (HepG2), or Dulbecco's modified Eagle's medium (ATCC) (293T cells), supplemented with 10% fetal bovine serum (Hyclone, Logan, UT, USA), L-glutamine and penicillin-streptomycin. The cells were grown in 12-well plates (6 × 10^4 ^cells/well for HepG2, 4 × 10^4 ^cells/well for 293T) and transfected using Fugene (Roche Molecular Biochemicals, Indianapolis, IN, USA) following the manufacturer's protocol. Briefly, 500 ng (for HepG2) or 100 ng (for 293T) of each assayed plasmid and 50 ng (for HepG2) or 10 ng (for 293T) pCMVβ (BD Biosciences, San Jose, CA, USA) were mixed with 1.5 μl Fugene and added to each well. Following 42 to 48 hours of incubation, cells were harvested and lysed. Activity of luciferase and β-galactosidase was measured using the Luciferase Assay System (Promega) and galacto-Light Plus (Applied Biosystems), respectively. Luciferase activity for each sample was normalized to the β-galactosidase assay control. Transfections were carried out in duplicates. All experiments are representative of at least three independent transfections.

### Tail vein plasmid DNA transfer assays

Tail vein injection was performed as described by Herweijer and Wolff [27] following the TransIT^® ^*In Vivo *Gene Delivery System Protocol (Mirus Corporation, Madison, WI, USA). Six to nine FVB male mice (Charles River Laboratory, Wilmington, MA, USA) at age 7 to 8 weeks were used for each reporter gene construct; 10 μg of each reporter construct, along with 2 μg of pCMVβ (BD Biosciences) to correct for delivery efficiency, were injected into each mouse. The entire content of the syringe was delivered in 3 to 5 seconds. Animals were sacrificed 24 hours later, livers extracted, measured to correct for size, homogenized, and centrifuged for 15 minutes at 4°C, 14,000 rpm. Activity of luciferase and β-galactosidase was measured as described above. All *p *values are from the two-sample Wilcoxon rank-sum (Mann-Whitney) test using STATA (STATA Corporation, College Station, TX, USA). All experimental results are representative of two independent plasmid DNA transfer assays.

### DNase I-hypersensitive site mapping

DNase I-hypersensitive site mapping was performed as described previously [28]. Briefly, cell pellets were resuspended in DNase I digestion buffer containing 0.5% IGEPAL (tert-Octylphenoxy poly(oxyethylene)ethanol) at 2 × 10^7 ^cells/ml buffer. Aliquots (100 μl) of the resuspended cells were mixed with equal volumes of DNase I buffer containing varying concentrations of DNase I.The DNase I digestion reaction was incubated at 23°C for 5 minutes before being stopped with the addition of 8 μl of 0.5 M EDTA and 2 μl of 100 mg/ml RNase A.Following 5 minutes of RNase A treatment, genomic DNA was isolated using the QiaQuickPCR Kit (Qiagen, Valencia, CA, USA). Then, 10 μg of each of the DNA samples was digested with an appropriate restriction enzyme and resolved in a 1.2% agarose gel by electrophoresis. The DNA from the gel was then transferred onto a nylon membrane. Southern blotting was carried out with a radiolabeled DNA probe generated by PCR amplification (see Additional data file 5 for primer sequences), and corresponds to regions of approximately 1,000 bp from Gumby predicted elements. Following hybridization and washing, the blot was exposed to Biomax film (Kodak Co., Rochester, NY, USA) with intensifying screens at -80°C for 48 hours.

## Additional data files

The following additional data are available with the online version of this paper.

Additional data file 1 is a figure showing sequence alignments of primate-conserved sequence LDLR_PS2. Additional data file 2 is a figure showing sequence alignments of primate-conserved sequence SREBF1_PS. Additional data file 3 is a figure documenting luciferase functional assays, which indicate enhancer activity for orthologous primate *LDLR *PS4 from baboon, dusky titi, marmoset and lemur. Additional data file 4 is a table listing the coordinates of evolutionarily conserved elements. Additional data file 5 is a table providing sequences of PCR primers used for cloning regulatory elements into reporter gene constructs or generating Southern blotting probes for detecting DNase I hypersensitive sites. Additional data file 6 is a table listing GenBank accession numbers for all primate BACs sequenced. Additional data file 7 contains the figure legends for Additional data files 1, 2, 3.

## Supplementary Material

Additional data file 1 Sequence alignments of primate-conserved sequence LDLR_PS2. Click here for file

Additional data file 2 Sequence alignments of primate-conserved sequence SREBF1_PS. Click here for file

Additional data file 3 Luciferase functional assays, indicating enhancer activity for orthologous primate *LDLR *PS4 from baboon, dusky titi, marmoset and lemur. Click here for file

Additional data file 4 Coordinates of evolutionarily conserved elements. Click here for file

Additional data file 5 Sequences of PCR primers used for cloning regulatory elements into reporter gene constructs or generating Southern blotting probes for detecting. DNase I hypersensitive sites Click here for file

Additional data file 6 GenBank accession numbers for all primate BACs sequenced. Click here for file

Additional data file 7 Figure legends for Additional data files 1, 2, 3. Click here for file

## Figures and Tables

**Table 1:** Functional characterization of non-coding elements significantly conserved only in primates

	*In vitro *(HepG2)		*In vivo *(mice)
	
Primate specific element	DNase I HS	Reporter transfection	Gene transfer
LDLR_PS1	No	No activity	No activity
LDLR_PS2	Yes	Enhancer* (~5.1-fold)	Enhancer (~5.5-fold)
LDLR_PS3	No	No activity	No activity
LDLR_PS4	Yes	Enhancer (~3.7-fold)	Enhancer (~4.2-fold)
SREBF1_PS	No	Silencer (~2.4-fold)	Silencer (~1.8-fold)
CYP7A1_PS	No	No activity	No activity

*In 293T cells. Human elements with primate-specific conservation were tested for their ability to drive reporter gene expression *in vitro *in HepG2 cells and *in vivo *in mouse liver. The genomic regions containing primate-conserved elements were also examined for the presence of DNase I hypersensitive sites (DNase I HS) in HepG2 cells. Enhancer or silencer strength is shown as fold increase or decrease relative to the promoter alone in luciferase assays.
