# Supplementary material for: Detection of weakly conserved ancestral mammalian regulatory sequences by primate comparisons
Source: Genome Biol. 2007 Jan 3;8(1):R1. doi: 10.1186/gb-2007-8-1-r1 (PMC1839124; doi:10.1186/gb-2007-8-1-r1)
Supplement: Additional data file 4 — Coordinates of evolutionarily conserved elements. [file gb-2007-8-1-r1-S4.doc]

**Table S1: Coordinates of evolutionarily conserved elements in the March 2006 human genome assembly,** hg 18.

| **Element ID Coordinates**  (Gumby prediction/cloned into reporter construct)  LDLR_PS1 chr19:11068178-11068454(277bp)/11067913-11068639(727bp)  LDLR_PS2 chr19:11110534-11110992(459bp)/11110333-11111194(862bp)  LDLR_PS3 chr19:11072133-11072436 (304bp)/11071911-11072685(775bp)    LDLR_PS4 chr19:11109355-11109741(387bp)/11109274-11110332 (1059bp)  SREBF1_PS chr17:17666279-17666547(269bp)/17665782-17667052(1271bp)  CYP7A1_PS chr8:59570766-59571183(418bp)/59570541-59571472(932bp) |
| --- |
