# Supplementary material for: Detection of weakly conserved ancestral mammalian regulatory sequences by primate comparisons
Source: Genome Biol. 2007 Jan 3;8(1):R1. doi: 10.1186/gb-2007-8-1-r1 (PMC1839124; doi:10.1186/gb-2007-8-1-r1)
Supplement: Additional data file 5 — Sequences of PCR primers used for cloning regulatory elements into reporter gene constructs or generating Southern blotting probes for detecting. DNase I hypersensitive sites [file gb-2007-8-1-r1-S5.doc]

**Table S2: PCR Primers used for cloning regulatory elements into reporter gene constructs (reporter) or generating southern blotting probes in detecting DNase I hypersensitive site (HS site). All elements are human sequences, unless indicated otherwise.**

| **Element ID Primers**  LDLR promoter (reporter) F: ATGCGTTTCCAATTTTGAGG  R: TCTAGCAGGGGGAGGAGTTT  LDLR_PS1 (reporter) F: AGCCTCAGTCATGCCACTG  R: GGCCTAGGCAACATACCAAG  LDLR_PS1 (HS site) F: GGTCTACCCAGTGGCACATT  R: GTGGGCAAAAAGCAATTGAG  LDLR_PS2 (reporter) F: ACTCCAGCCTGGGAAACTCT  R: GGAGGCCACTGTGTCAGTTT  LDLR_PS2 (HS site) F: CCACAAAACCAGGAGAGGAA  R: CTCCTTCGATCCACCCAGTA  LDLR_PS2_Mouse (reporter) F: GCAGCAGCTGATTTCTGACA  R: AGGCATGCTTGTGAGAGGTA  LDLR_PS3 (reporter) F: GCGAAAGAAACGAGTTCCAG  R: CAGGTGGATCACGAGGTCA  LDLR_PS3 (HS site) F: GCAAGGCTGTCGTAAGTGTG  R: CTGACTGTGCGTGACAAACC  LDLR_PS4 (reporter) F: ACTCCAGCCTGGGAAACTCT  R: GGAGGCCACTGTGTCAGTTT  LDLR_PS4 (HS site) F: CCACAAAACCAGGAGAGGAA  R: CTCCTTCGATCCACCCAGTA  LDLR_PS4_Mouse (reporter) F: CTTGGGGAAACAGAACAGGA  R: GTGAGTTGGAAGGGACCACA  LDLR_PS4_baboon (reporter) F: GCTAGCCTGAGATCCCGCCATTGT  R: GGTACCCTCCCACCTTCCACTCTGTC  LDLR_PS4_dusky titi (reporter) F: GCTAGCGGCGCATGCCTGTAATCT  R: GGTACCTTAGCCTGGGGTGGTGGT  LDLR_PS4_marmoset (reporter) F: GCTAGCtcacttgaatccaggaggtg  R: GGTACCggatcagaaaccagccacat  LDLR_PS4_lemur (reporter) F: ACTAGTAATAGCTGGGTGTGGTGGTC  R: GGTACCCAGCCCTCACTCTGTCTGC  SREBF1c promoter (reporter) F: GAGTTCTGGTGTGTTGGGCCA  R: CCGCGCTGCCGCCTCGCTAG  SREBF1_PS (reporter) F: GAGAATGGCAGACAGGGAAG  R: GGAAACCCTGCAGTTGAAAG  SREBF1_PS (HS site) F: GTGGACTCAGACCAGCTTCC  R: GAGCTGCTGGATCTGTGGAT  SREBF1_PS_Mouse (reporter) F: CTAACGGCCTCTGAGAGTGG  R: GGCATGAGAGATGGAAGGTC    CYP7A1 promoter (reporter) F: TAGCTATGCCCATCTTAAACAGGTT  R: AATCTCTGAGGAAGAAAATCTCTGA  CYP7A1_PS (reporter) F: CCGTGTTAGCCAGGATGG  R: AAGCCACGGAGCTTGTATGT  CYP7A1_PS (HS site) F: CAGGGCTGGAAAGACTTTGT  R: ATGATGGAAAACCTCCAACG |
| --- |
