# Supplementary material for: Detection of weakly conserved ancestral mammalian regulatory sequences by primate comparisons
Source: Genome Biol. 2007 Jan 3;8(1):R1. doi: 10.1186/gb-2007-8-1-r1 (PMC1839124; doi:10.1186/gb-2007-8-1-r1)
Supplement: Additional data file 6 — GenBank accession numbers for all primate BACs sequenced. [file gb-2007-8-1-r1-S6.doc]

**Table S3: GenBank accession numbers for all primate BACs sequenced.**

| Baboon Colobus Marmoset Dusky titi Owl* Squirrel** Lemur |
| --- |
| LDLR AC140974 AC150433 AC145530 AC144655 AC171393 --- AC118569  SREBF1 AC147417 AC148225 AC148170 AC147424 AC147425 AC151885 AC141085  CYP7A1 AC162431 AC148223 AC162435 --- AC162781 AC147423 AC151871  LXRA AC140973 AC148224 --- AC146902 AC187542 AC187541 AC118575  ABCG5/8 AC151855 AC150375 AC146466 AC146286 AC146787 AC146464 AC145533  APOE AC145523 AC148222 AC146283 AC146285 AC146520 AC151887 AC135911  APOA5 AC145521 AC148228 AC145529 AC144989 AC146499 AC146293 AC118574  HMGCR AC150373 AC150434 AC150377 AC150381 AC150439 AC150438 AC150436 |

*Owl: owl monkey. **Squirrel: squirrel monkey.

baboon (*Papio hamadryas*), colobus (*Colobus guereza*), marmoset (*Callithrix jacchus*), dusky titi (*Callicebus moloch*), owl monkey (*Aotus hybrid*), squirrel monkey (*Saimiri boliviensis boliviensis*), lemur (*Lemur catta*).

---: insufficient sequences.
