# Supplementary material for: Detection of weakly conserved ancestral mammalian regulatory sequences by primate comparisons
Source: Genome Biol. 2007 Jan 3;8(1):R1. doi: 10.1186/gb-2007-8-1-r1 (PMC1839124; doi:10.1186/gb-2007-8-1-r1)
Supplement: Additional data file 7 — Figure legends for Additional data files 1, 2, 3. [file gb-2007-8-1-r1-S7.doc]

**Supplemental Figure Legends**

**Fig.S1: Evolutionary conservation of 6 primate-conserved sequence in anthropoid primates, but not between human and mouse or dog.** Sequence alignment of primate-conserved sequence LDLR_PS2 (A) and SREBF1_PS (B).

**Fig. S2** **Functional assays indicate enhancer activity for orthologous primate *LDLR* PS4.** Luciferase assay analysis of transient transfections into human HepG2 cells. The luciferase reporter constructs tested are either the LDLR promoter alone (promoter), or the promoter in combination with indicated primate LDLR PS4 (+ PS4). Fold increase over the empty vector is shown. Error bars indicate standard deviation.
